# Enhancement of Ultrasound B-Mode Image Quality Using Nonlinear Filtered-Multiply-and-Sum Compounding for Improved Carotid Artery Segmentation

**DOI:** 10.3390/diagnostics13061161

**Published:** 2023-03-18

**Authors:** Asraf Mohamed Moubark, Luzhen Nie, Mohd Hairi Mohd Zaman, Mohammad Tariqul Islam, Mohd Asyraf Zulkifley, Mohd Hafiz Baharuddin, Zainab Alomari, Steven Freear

**Affiliations:** 1 Department of Electrical, Electronic and Systems Engineering, Faculty of Engineering and Built Environment, Universiti Kebangsaan Malaysia, Bangi 43600, Selangor, Malaysia; 2 School of Electronic and Electrical Engineering, University of Leeds, Leeds LS2 9JT, UK; 3 Department of Communications Engineering, Electronics Engineering College, Ninevah University, Mosul 41002, Iraq

**Keywords:** biomedical, ultrasound B-mode imaging, filtered multiply and sum, filtered-delay multiply and sum, active contour, common carotid artery

## Abstract

In ultrasound B-mode imaging, the axial resolution (AR) is commonly determined by the duration or bandwidth of an excitation signal. A shorter-duration pulse will produce better resolution compared to a longer one but with compromised penetration depth. Instead of relying on the pulse duration or bandwidth to improve the AR, an alternative method termed filtered multiply and sum (FMAS) has been introduced in our previous work. For spatial-compounding, FMAS uses the autocorrelation technique as used in filtered-delay multiply and sum (FDMAS), instead of conventional averaging. FMAS enables a higher frame rate and less computational complexity than conventional plane-wave compound imaging beamformed with delay and sum (DAS) and FDMAS. Moreover, it can provide an improved contrast ratio and AR. In previous work, no explanation was given on how FMAS was able to improve the AR. Thus, in this work, we discuss in detail the theory behind the proposed FMAS algorithm and how it is able to improve the spatial resolution mainly in the axial direction. Simulations, experimental phantom measurements and in vivo studies were conducted to benchmark the performance of the proposed method. We also demonstrate how the suggested new algorithm may be used in a practical biomedical imaging application. The balloon snake active contour segmentation technique was applied to the ultrasound B-mode image of a common carotid artery produced with FMAS. The suggested method is capable of reducing the number of iterations for the snake to settle on the region-of-interest contour, accelerating the segmentation process.

## 1. Introduction

Improving the axial resolution (AR) will bring many benefits for numerous applications in ultrasound imaging, such as localizing single microbubbles for super-resolution, segmenting the cross-sectional area of a carotid artery for stenosis assessment and measuring the intima-media thickness of a common carotid artery for diagnosing cardiovascular diseases [1,2,3,4,5,6]. Numerous methods have been proposed to improve the AR in ultrasound B-mode imaging. They include the use of wideband excitation signals and the employment of pre/post signal processing techniques [7,8,9,10]. Short excitation pulses, such as a broadband square pulse signal, are able to produce a narrow main lobe along the axial direction. However, the side lobe produced by the excitation signal is also relatively very high due to spectral leakage from the diffraction effect [7,11]. When the side-lobe energy of a strong scatterer exceeds the main lobe energy of an adjacent weak scatterer, the weak scatterer becomes easily obscured or distorted. Therefore, developing a powerful side-lobe reduction method is crucial [12]. The high side lobe is the main contributor to a low image contrast ratio (CR) [13]. Moreover, the penetration depth with the square pulse is considerably shallow because the acoustic attenuation in the tissue increases with the imaging depth and the excitation signal frequency. Increasing the square pulse energy level may not be the best solution to improve the imaging penetration depth. This is due to the limitation set by the Food and Drug Administration (FDA) that requires the acoustic pressure to be within the safety limit [7,14]. This is done to ensure that the acoustic intensities will not cause thermal and mechanical damage to the imaged tissue. Thus, to improve the penetration depth and the AR, researchers have turned to chirp-pulse-coded excitation signals.

The long pulse duration of the chirp signal combines two important features which are high energy with a low mechanical index and a broadband bandwidth [7]. A chirp signal is able to produce good AR and penetrate deeper into the imaging medium. Employing chirps or coded excitation signals requires a matched or mismatched filter during the receive beamforming stage to compress the echoes [7]. Pulse compression is a method used to restore the AR when using a chirp excitation signal. One of the main drawbacks of this method is the trade-off between the AR and CR of the B-mode image. A matched filter can produce a better AR with a low CR, while a mismatched filter is able to reduce the side lobe at the cost of broadening the main axial lobe. Sacrificing one metric to improve another is not the best solution for ultrasound B-mode imaging. In [15], the authors highlighted the importance of improving the AR for enhanced endoscopic ultrasound imaging. However, their compression pulse weighting method (CPWM) applied to the chirp excitation signal was not able to improve the AR. The CPWM approach produced a 0.13 mm main lobe at −20 dB, while the sine wave and matching filter both achieved 0.12 mm.

A new beamforming technology named filtered-delay multiply and sum (FDMAS) was introduced in 2014 by Giulia Matrone et al. [16]. It could provide a better contrast than conventional delay and sum (DAS) and took less time to compute than minimum variance. However, the improvement in AR was very minimal when compared to DAS. This could be seen in multiple publications [16,17,18]. Qualitatively, no significant changes could be seen from the lateral and axial beam profiles at the full width at half-maximum (FWHM) and −20 dB. However, the numerical results presented by Giulia Matrone et al. showed that FDMAS was able to improve the AR by approximately 10% compared to DAS. The two most common compounding techniques used in plane-wave imaging with DAS and FDMAS beamformers are coherent and noncoherent averaging [19,20]. Compounding is used to reduce clutter noise after beamforming for each plane-wave transmission. This is because the side lobe of the target occurs at different spatial locations, and they are loosely correlated for different plane-wave transmissions. This phenomenon happens because each plane wave during transmission is assigned a unique time delay. Meanwhile, the primary lobe of the target position remains constant and is significantly correlated with steered plane waves [21]. However, with conventional compounding technique, the noise reduction inside the anechoic region is minimal. Thus, a high number of plane waves with large steering angles are required to produce a high-contrast B-mode image with a low frame rate [22]. The challenge of acquiring a good-quality B-mode image of a moving object is undeniable. Thus, using a limited number of compounding angles to achieve high frame rates is mandatory. This may not be achievable with the conventional compounding technique.

In our previous work [23], we proposed a nonlinear compounding technique known as filtered multiply and sum (FMAS). However, we just gave a brief introduction of the concept and did not explain in detail the potential applications and main theory behind the improvement of AR. Several researchers [24,25,26,27,28] also implemented and compared our proposed method, but the results they obtained and presented had several flaws. First, the dynamic range used to present all the B-mode images in their work was 70 dB, which was very high compared to the conventional range of 40 to 60 dB. Displaying the B-mode image with a high dynamic range (≥60 dB) will include all clutter noise that is present in the B-mode image processed with DAS thus making it qualitatively look like a very low quality image. If the same B-mode image is presented with a dynamic range of 40 to 60 dB, it may appear better in terms of a high-contrast image, though no changes in spatial resolution can be achieved. Second, the improvements in spatial resolution and noise reduction achieved by other researchers were still comparably low with what we present in this work. This can be due to the improper selection of the band-pass filter (BPF) after the autocorrelation process. The selection of the BPF will determine whether only the frequencies of interest are extracted, or noise is also included.

Image enhancement is of great importance in biomedical image segmentation. Image segmentation is a crucial step in establishing a solid foundation for clinical diagnostics, such as 3D anatomical reconstruction and image analysis and visualization [29,30,31]. Active contour is a popular segmentation approach for separating desired regions from a variety of biomedical images [32,33,34]. Thus, in this study, balloon-snake active-contour (BSAC) segmentation is performed on the common carotid artery B-mode image to prove that the AR enhancement with the proposed FMAS method can reduce the number of iterations and computational time during the segmenting process. Less clutter noise and a good image resolution are two main criteria for a better segmentation [22]. In this work, we discuss in detail the theory behind the FMAS algorithm and how it is able to improve the spatial resolution mainly in the axial direction with as few as three plane waves. The low complexity of the proposed method also means it has a high potential to be implemented for real-time imaging. We also demonstrate how FMAS may be used in a practical biomedical image segmentation application.

## 2. Methods and Materials

### 2.1. Coherent Plane-Wave Imaging (PWI)

To efficiently improve the frame rate, unfocused plane waves have been proposed to insonify the whole region of interest (ROI), where the ultrasound image is reconstructed by revisiting the same set of received radio frequency (RF) channel data when beamforming each image pixel. A number of plane-wave transmissions are usually used, and both the resolution and contrast of the final image can be improved by compounding the low-quality images beamformed for individual plane-wave transmissions. Details about ultrasound beamforming and plane-wave imaging can be found elsewhere, and readers are referred to the following publications [19,35].

It requires a total of *N* steered plane waves (*n*) to attain a comparable quality as a focused image at *z* mm depth [20],

(1) $$N=\frac{L_{a}}{\lambda F}=\frac{L_{a}^{2}}{\lambda_{z}}$$

where *F* is the F-number calculated as $z/L_{a}$, $L_{a}$ is the aperture’s length, λ is the signal wavelength, and $\theta_{n}$, the steering angle, is given by:

(2) $$\theta_{n}=\arcsin\left(\frac{n\lambda }{L_{a}}\right)\approx \left(\frac{n\lambda }{L_{a}}\right)$$

where *n* is defined as

(3) n=−(N−1)/2,...,(N−1)/2

The primary focus of this research was to investigate how the compounding approach, FMAS, affected imaging results when employing different numbers of compounding angles with the DAS and FDMAS beamformers, rather than to determine the optimum number of compounding angles. Based on their experimental settings, numerous studies have proposed a specific number of compounding angles as having the best image quality [19,20,21]. Image quality does not improve beyond a certain number of compounding angles, according to several studies [19,21], but instead degrades due to poor noise suppression near the main lobe. These limitations were taken into consideration when presenting the selected number of compounding angles, *N*, and the steering angle increment, $\Delta \theta^{\circ }$ in Table 1. The sector angles, $\left[\theta_{max}^{\circ },\theta_{min}^{\circ }\right]$ and $\pm 12^{\circ }$ were set for all compounding conditions.

### 2.2. DAS and FDMAS Beamforming

Beamforming is one of the most important steps in ultrasound B-mode imaging used to reconstruct the received echo from the imaged medium. The initial steps of DAS and FDMAS are identical. The RF signal that each element, *i*, receives in this case is the signal, $s_{i}\left(t\right)$. To calculate the required focusing delay, $\tau_{i}$, to temporarily align the signals received by each element, the following equation is used:

(4) $$\tau_{i}\left(x,Z\right)=\frac{(Z\cos\theta_{n}+x\sin\theta_{n}+\frac{L_{a}}{2}\sin\theta_{n}}{c}+\frac{\sqrt{Z^{2}+\left(x_{i}-x^{2}\right)}}{c}$$

where *x* is the lateral location of the beamformed pixel with a step size of λ/3, and Z is the vector of axial pixel locations given by

(5) $$Z=\left[\begin{array}{c} z_{1}\\ z_{2}\\ \vdots \\ z_{depth} \end{array}\right]$$

*c* is the sound speed, $z_{depth}$ is the maximum imaging depth, and $x_{i}$ is the distance between the *i*th element and the centre of the transducer. The aligned RF signal, $v_{i}\left(x,Z\right)$, is the RF signal with the focusing delay compensated, $s_{i}\left(x,Z\right)$, and it can be expressed by the following equation:

(6) $$v_{i}\left(x,Z\right)=s_{i}\left(t-\tau_{i}\left(x,Z\right)\right)$$

The aligned signals in DMAS, as opposed to DAS, undergo a procedure similar to autocorrelation, which is denoted by the equation:

(7) $$r_{DMAS}\left(x,Z\right)=\sum_{i=1}^{E-1}\sum_{m=i+1}^{E}sgn\left\{v_{i}\left(x,Z\right)v_{m}\left(x,Z\right)\right\}\times \sqrt{\left(|v_{i}\left(x,Z\right)v_{m}\left(x,Z\right)\right|}$$

where m=i+1 is the aligned RF signal at the *m*th element and *E* is the total number of elements on the imaging probe. The second-harmonic and direct-current (DC) components are created by multiplying two RF signals with the same frequency. In order to extract the second-harmonic from $r_{DMAS}\left(x,Z\right)$, a BPF is used. As a result, $r_{FDMAS}\left(x,Z\right)$ is produced. To create a vertical imaging line, *l*, at a specified lateral location *x*, a number of time delays are determined for each depth Z.

### 2.3. Filtered-Multiply-and-Sum (FMAS) Compounding

Following DAS beamforming for individual steered plane waves, the FMAS compounding procedure is performed. The beamformed RF frames are autocorrelated to generate the multiply-and-sum (MAS) frames, as opposed to the traditional compounding process, which involves adding and averaging all the steered plane waves after beamforming. The following is the MAS equation:

(8) $$Com_{MAS}=\sum_{n=}^{N-1}\sum_{k=n+1}^{N}sgn\left\{V_{n}\left(t\right)V_{k}\left(t\right)\right\}\times \sqrt{|V_{n}\left(t\right)V_{k}\left(t\right)|}$$

where V(t) denotes a collection of aligned RF signals $v_{i}\left(t\right)$ (i= 1 to 128) for each directed plane wave. The method is comparable to how the autocorrelation function works. The proposed method is faster than FDMAS because the number of multiplications, *B*, involved in the autocorrelation for FMAS is equal to the number of compounding angles of *N* as indicated by

(9) $$B=\frac{N^{2}-N}{2}$$

To generate a filtered-multiply-and-sum (FMAS) compounded signal, the RF signals obtained from MAS must be band-pass-filtered. The explanation is the same as in FDMAS, where two separate frequency spectra (DC and second-harmonic) are formed by multiplying two RF signals with the same frequency [16] (the 6th figure in Section 3 exhibits the related frequency components). The RF signals are then filtered, Hilbert transformed for envelope detection and log-compressed to generate the B-mode image.

### 2.4. Active Contour Segmentation

The user-defined approximation of an object’s border or contour serves as the starting point for active-contour-based segmentation techniques [36,37,38]. The initial contour then evolves, establishing the actual object boundaries. The aim of active contour generation is to evolve continuously over a predefined number of iterations in order to minimise the overall external energy (image gradient) and internal energy (contour form) [39,40]. The total snake energy is calculated so that it is always at its minimum at the end of each iteration. By resolving the following Euler–Lagrangian equation, it is possible to minimise the total snake energy using the calculus of variation [39]:

(10) $$\frac{\delta }{\delta_{S}}\left(\alpha \left(s\right)V_{s}\left(s\right)\right)+\frac{\delta^{2}}{\delta_{S}^{2}}\left(\beta \left(s\right)V_{s}\left(s\right)\right)-\nabla E_{ext}\left(V(s)\right)=0$$

where the points on the contour, V(s), are represented by s∈[0,1]. The first derivative, $V_{s}\left(s\right)$, gives a measure of the contour control’s elasticity (stretching) strength via α(s), whereas the second derivative, $V_{ss}\left(s\right)$, offers a measure of the stiffness (bending) strength of the contour control via β(s). The external energy of an image is represented by $E_{ext}\left(V\left(s\right)\right)$.

### 2.5. Simulation and Experimental Setup

To validate the FMAS compounding method, various measurements were done on point targets (wire) with a diameter of 120 μm in deionized and degassed water, on a tissue-mimicking phantom (040GSE, CIRS, Norfolk, VA, USA) and in vivo. Anechoic segments (10 mm to 50 mm in depth) and point targets (10 mm to 60 mm in depth) of the tissue-mimicking materials were scanned. A right common carotid artery cross section of a healthy volunteer was used to collect in vivo data. A linear array transducer of 128 elements (L3-8/40EP, Prosonics Co. Ltd., Gyeongju-si, Republic of Korea) with a 4.79 MHz centre frequency and a 57% bandwidth at −6 dB was used to collect all the data. The multipurpose imaging system, ultrasound array research platform II, (UARP II, University of Leeds, Leeds, UK), stimulated the transducer with a two-cycle sinusoidal wave signal with a centre frequency of 5 MHz [41,42,43]. The signals received were sampled at a frequency of 80 MHz. Table 2 contains all of the simulation and experimental parameters.

### 2.6. Performance Evaluation

The quality of the final B-mode images obtained by the DAS, FDMAS and DAS-FMAS approaches can be evaluated using two key metrics: spatial resolution and CR. The function developed in [44] was used to measure the primary lobes of the point target, a nylon wire with a diameter of 120 μm placed at a depth of 30 mm in deionized and degassed water at −6 dB and −20 dB in order to calculate the AR and lateral resolution (LR). A cyst’s detectability was expressed using the CR by comparing values between the cyst’s ROI and its background. The contrast-to-noise ratio (CNR) in ultrasound images was used to quantify the cyst’s contrast and detectability. In order to measure the CNR and CR, the B-mode image of a 3.0 mm diameter cyst with anechoic fluid located at a depth of 15 mm was generated using the 040GSE phantom by creating two identically sized sections. The first area was established inside the cyst, and the second area was established at the same depth outside the cyst. This requirement ensures that the attenuation with depth would not have an effect on the measurements. The following are the CR and CNR equations: [22,45],

(11) $$\mathrm{CR}(\mathrm{dB})=20log_{10}\left(\frac{\mu_{cyst}}{\mu_{back}}\right)$$

(12) $$\mathrm{CNR}(\mathrm{dB})=20log_{10}\left(\frac{|\mu_{cyst}-\mu_{back}|}{\sqrt{(\sigma_{cyst}^{2}+\sigma_{back}^{2}}}\right)$$

where the means of the image intensities inside and outside the cyst are $\mu_{cyst}$ and $\mu_{back}$, respectively, and their variances are $\sigma_{back}^{2}$ and $\sigma_{cyst}^{2}$. All simulation and experiment findings were reported as a mean value with one standard deviation, determined from 10 measurement runs. During the repeated measurements, the transducer was not moved along the elevation axis.

The Dice coefficient and mean intersection over union (MIoU) metrics were used to assess the performance of the BSAC segmentation for a tissue-mimicking phantom. The Dice coefficient is the evaluation index most often used in segmentation. The higher the Dice coefficient, the greater the similarity between two samples. The MIoU determines how closely the two sets of elements overlap. The overlap area between the estimated segmentation and the ground truth is compared to the combined area of the two. The Dice and MIoU equations are as follows [46,47]:

(13) $$\mathrm{Dice}(A,B)=\frac{2|\left(A\cap B\right)|}{\left|A|+|B\right|}$$

(14) $$\mathrm{MIoU}(A,B)=\frac{\left|A\cap B\right|}{\left|A\cup B\right|}$$

where A represents the estimated segmentation and B represents the ground truth.

## 3. Results and Discussion

### 3.1. Simulation Results

**Figure 1:**
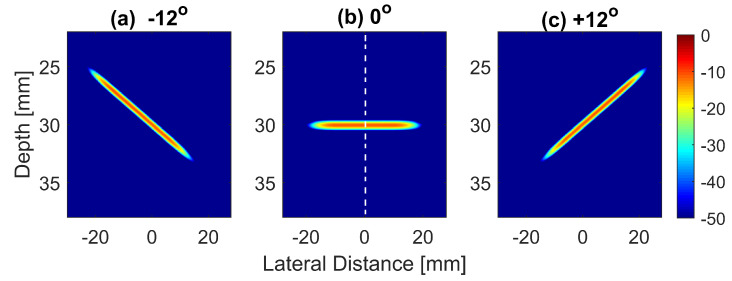
Normalized and log-compressed pressure fields for plane waves steered at (**a**) $-12^{\circ }$, (**b**) $0^{\circ }$ and (**c**) $+12^{\circ }$. A transmit apodization with a Tukey window (α=0.5) was applied to all emitted pressure fields in order to eliminate the edge waves on both sides of the field.

It was expected that with spatial compounding, the LR would be improved. This was due to the side lobe cancellation in the lateral direction. With both DAS and FDMAS, the LR increased with the number of compounding angles, but there were only small changes in the AR. This was because the AR was mainly determined by the bandwidth of the excitation signal regardless of the beamforming techniques or the number of compounding angles. However, with the proposed FMAS compounding technique, the AR was improved significantly when compared to that with DAS and FDMAS. This was explained by the concept that the geometry and appearance of the objects in PWI were influenced by the beam concentration or directivity. Steered plane waves had distinct intensity distributions for each angle. The beam pattern and its intensity distribution were changed in accordance with the angle’s increase or decrease. This phenomenon was mostly visible on the lateral side lobes and the axial lobes in the axial direction, where it appeared at different locations depending on the steering angles. In order to analyse this phenomenon in detail, Field II simulations were performed to obtain the emitted pressure fields for different steering angles at a 30 mm depth. The setup for the simulation is given in Table 2. The emitted pressure fields simulated for steering angles −$12^{\circ }$, $0^{\circ }$ and $+12^{\circ }$ are shown in Figure 1.

**Figure 2:**
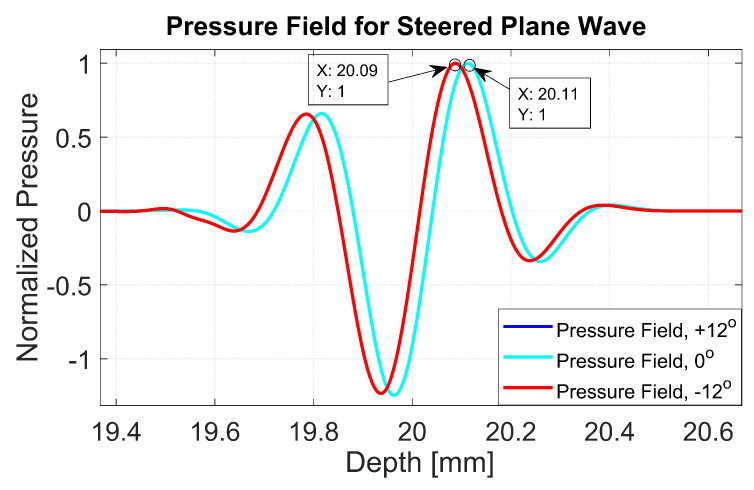
Normalized pressure fields at the position of the dashed line as shown in Figure 1 for plane waves steered at $-12^{\circ }$, $0^{\circ }$ and $+12^{\circ }$. The pressure fields for steering angles $-12^{\circ }$ and $+12^{\circ }$ overlap with each other.

**Figure 3:**
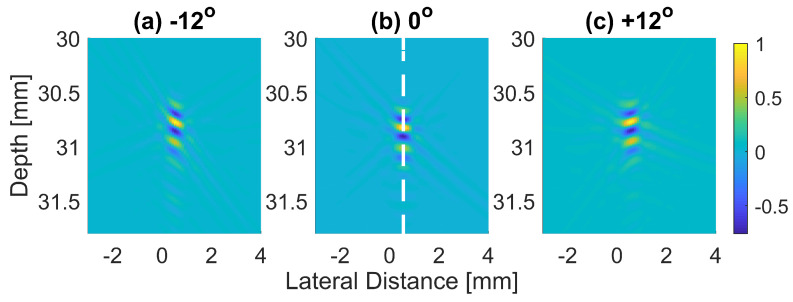
The received RF signals beamformed with DAS (before envelope detection and log compression) for a wire target with plane waves steered at (**a**) $-12^{\circ }$, (**b**) $0^{\circ }$ and (**c**) $+12^{\circ }$.

**Figure 4:**
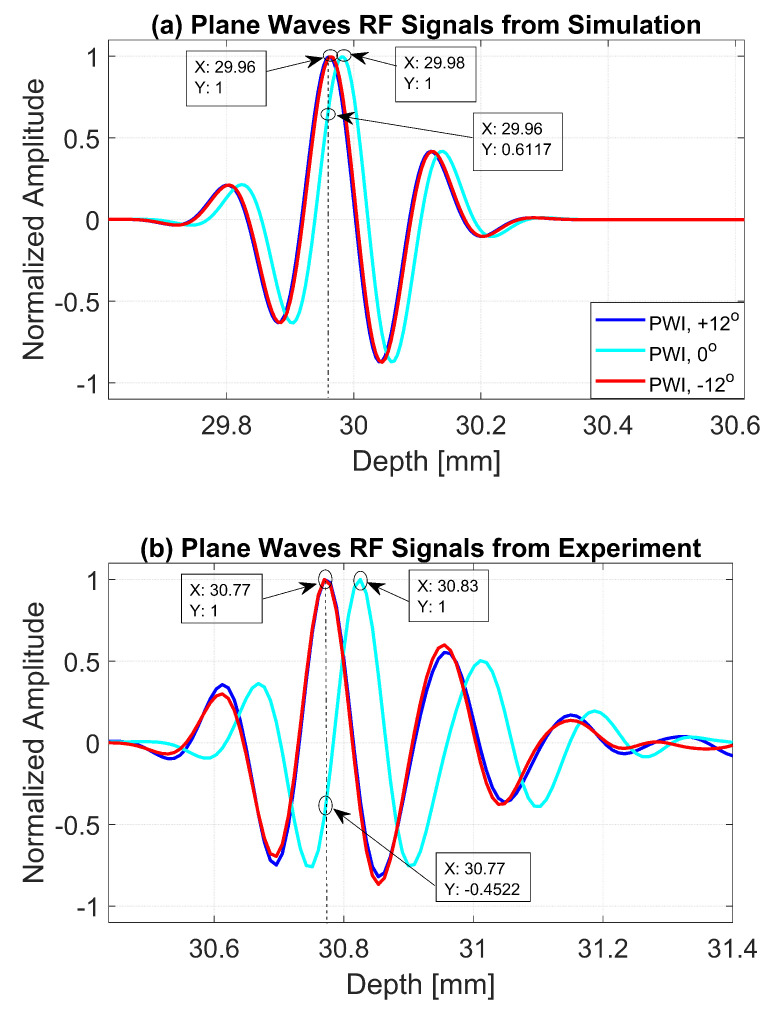
RF signals for the dashed line as shown in Figure 3b from plane waves steered at $-12^{\circ }$, $0^{\circ }$ and $+12^{\circ }$ with (**a**) Field II simulations and (**b**) experimental measurements.

The normalized pressure fields at x=0 mm as highlighted by the dashed line in Figure 1b are shown in Figure 2 for the three steering angles. The variations between pressure fields steered at $\pm 12^{\circ }$ and $0^{\circ }$ in the axial direction were clearly visible. There was a phase shift of 0.02 mm between peak pressure points between those steering angles. While the shift was invisible between plane waves steered at $-12^{\circ }$ and $+12^{\circ }$ since both steered pressure waves appeared at the same spatial location. The simulation to measure the phase shift between the pressure points was repeated on the point target located at 30 mm depth. Figure 3 shows the received RF signals for a wire target with plane waves steered at $\pm 12^{\circ }$ and $0^{\circ }$. The 0.02 mm shift between RF peaks was also found in the axial direction for the point target between steering angles of $+12^{\circ }$ and $0^{\circ }$. This is shown in Figure 4a. However, this shift was 0.06 mm for the experimental point target between steering angles of $+12^{\circ }$ and $0^{\circ }$. This is shown in Figure 4b. Even though the variation was too small to be considered in conventional compounding techniques, which apply averaging between the steered plane waves, this was not the case when a procedure similar to autocorrelation was used in the proposed technique. For example, when averaging three points as shown in Figure 4a, it produced an amplitude value of 0.87, while applying a procedure similar to autocorrelation as given by Equation (8) for the same three points produced an amplitude value of 1.39. Even a 0.02 mm variation between the aligned RF signals produced a significant difference in the main lobe values in the axial direction when FMAS compounding was applied. Further implications of the phase shift in the RF signals in the axial direction could be seen from the experimental result. Due to many other factors such as phase aberration, the variation between the RF signals further increased up to 0.06 mm. There, as shown in Figure 4b, the steering effect caused the RF signals appearing at x= 30.77 mm to have the amplitude values of 1 and −0.4522 for steering angles $\pm 12^{\circ }$ and $0^{\circ }$, respectively.

**Figure 5:**
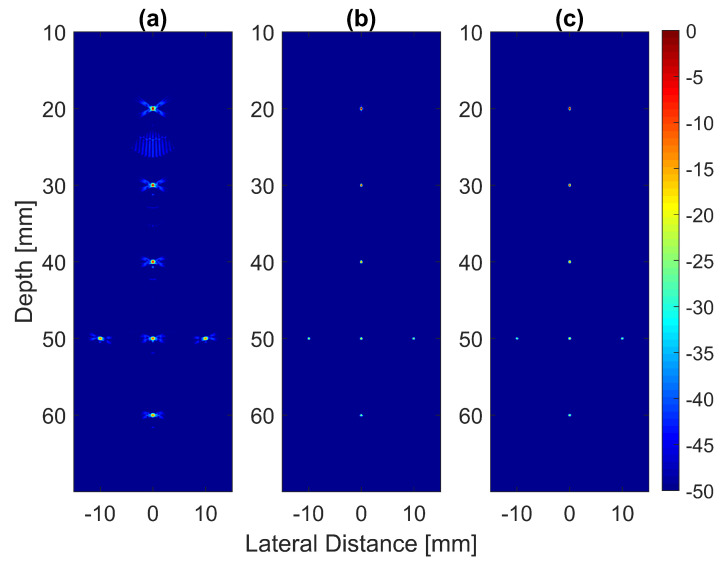

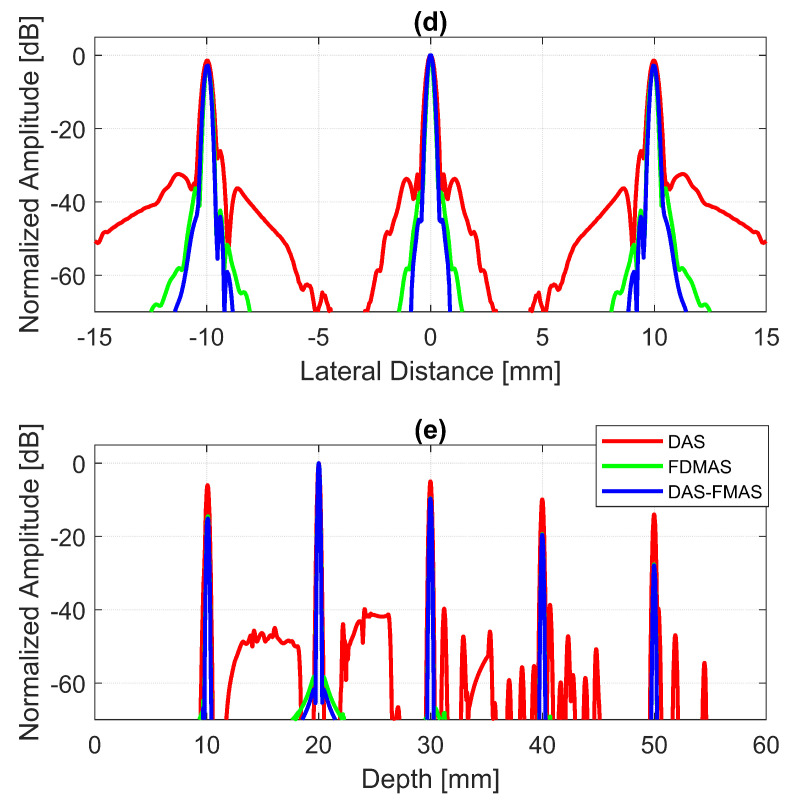
Point targets compound PWI B-mode images beamformed with (**a**) DAS, (**b**) FDMAS and (**c**) DAS-FMAS, N=3 ($-12^{\circ }$, $0^{\circ }$, $+12^{\circ }$). The lateral beam profile at the depth of 50 mm and the axial beam profile at x=0 mm are shown in (**d**,**e**) for all three beamforming techniques (DAS, FDMAS and DAS-FMAS).

**Figure 6:**
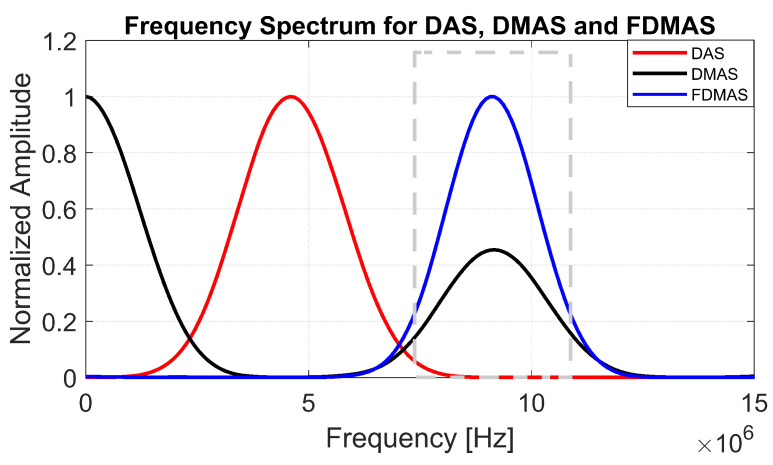
Normalized frequency spectra obtained with DAS, FDMAS and FMAS. The frequency spectrum was performed on a single point target located at a 30 mm depth as shown in Figure 3. The dashed grey box represents a BPF between 8.5 and 11.5 MHz to extract the second-harmonic component for DMAS.

The B-mode images obtained from Field II were processed for seven point targets located from a 10 mm to a 60 mm depth, and they are shown in Figure 5a–c. The corresponding beam patterns along the lateral direction at a z=50 mm depth and the axial direction at x=0 mm for DAS, FDMAS and DAS-FMAS are shown in Figure 5d, and Figure 5e, respectively. The proposed new compounding technique, FMAS, was able to eliminate the grating lobes that appeared at the 10 mm depth in both lateral directions (−10 mm and 10 mm) when beamformed with DAS as shown in Figure 5a. The new compounding method also reduced axial and side lobe in both axial and lateral directions. This is shown in Figure 5d,e. Up to 7 dB of peak side lobe (PSL) along the lateral direction was reduced with the new compounding technique, although FDMAS and FMAS used the same mathematical theorem, a process similar to autocorrelation. The beam pattern produced along the axial direction with FMAS was almost the same as that using FDMAS. The signal intensity level with FMAS was lower than that with DAS except at the depth of elevation focus. This was because all signals had been normalized to their maximum value. The explanation for this phenomenon was the same as what happened with FDMAS. When RF signals with almost identical frequency components from two steering angles were multiplied, DC and second-harmonic components were produced. The second-harmonic component, as shown in Figure 6 with a lower amplitude level, was used to form all images in DAS-FMAS.

**Figure 7:**
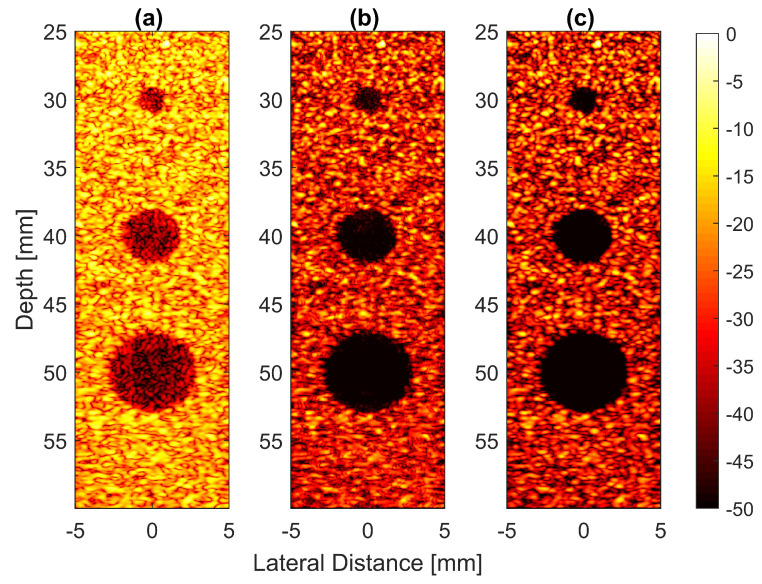

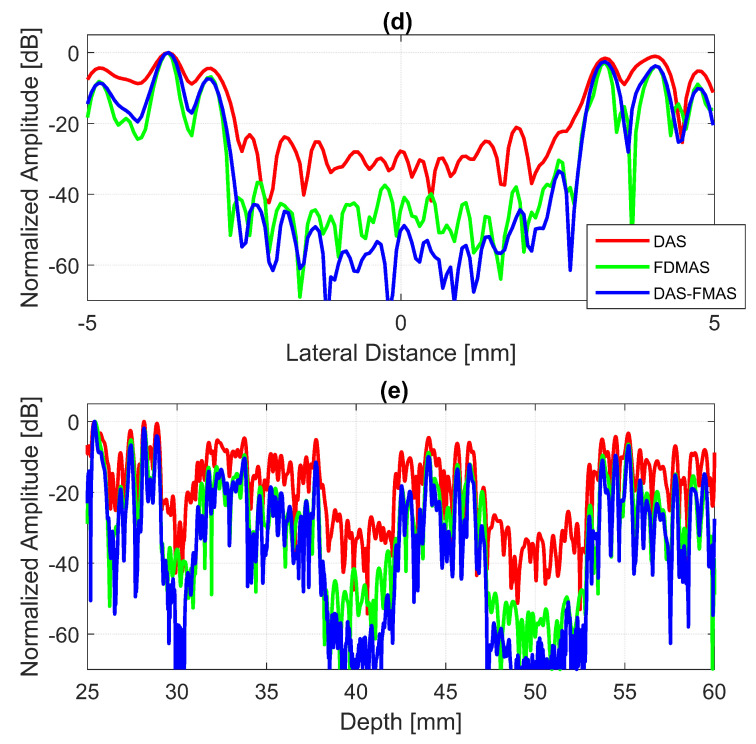
Field II simulations were performed on cysts located at depths of 30 mm, 40 mm and 50 mm with diameters of 2 mm, 4 mm and 6 mm with (**a**) DAS, (**b**) FDMAS and (**c**) DAS-FMAS. The number of steering angles was N=3 ($-12^{\circ }$, $0^{\circ }$, $+12^{\circ }$). (**d**) Lateral beam profiles for the 6 mm cyst at a 50 mm depth and (**e**) axial beam profiles along x=0 mm.

Thus, the signal had a lower intensity level. Low-signal intensities at a deeper location could be amplified by applying time gain compensation (TGC). Axial lobes (ALs) that occurred when plane waves were steered were visible below the point targets located at the depths of 10 mm and 20 mm, as shown in Figure 5a. Both FDMAS and FMAS were able to reduce these ALs. The spatial distributions of ALs for different steering angles were diverse, thus, when a process similar to autocorrelation took place, the decorrelation between the ALs was higher, making more ALs attenuated. The ALs mainly occurred at around the −45 dB level with DAS and were attenuated to below −60 dB with FDMAS and DAS-FMAS, as shown in Figure 5e. The CR for the simulated cyst located at the 30 mm depth was significantly improved with DAS-FMAS with just three steering angles ($-12^{\circ }$, $0^{\circ }$ and $+12^{\circ }$). Although FDMAS was able to improve the CR, clutter noise inside the anechoic region was still visible and not fully eliminated. The border definition for all cysts was improved with FDMAS, but more improvement was obtained with DAS-FMAS due to the further reduction of clutter noise. This can be seen clearly in Figure 7. The attenuation of clutter noise inside the cyst region was because lateral side lobes leaking into the anechoic region were significantly reduced, thus making the edge steeper and improving the border definition.

**Figure 8:**
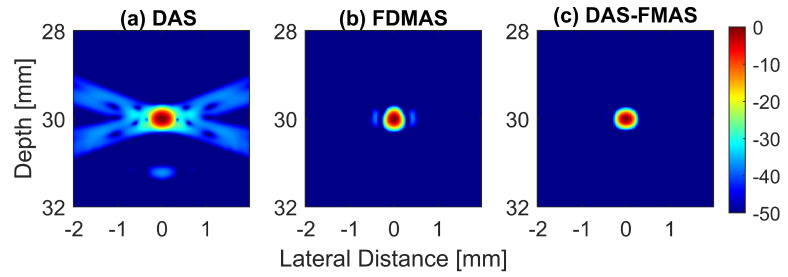

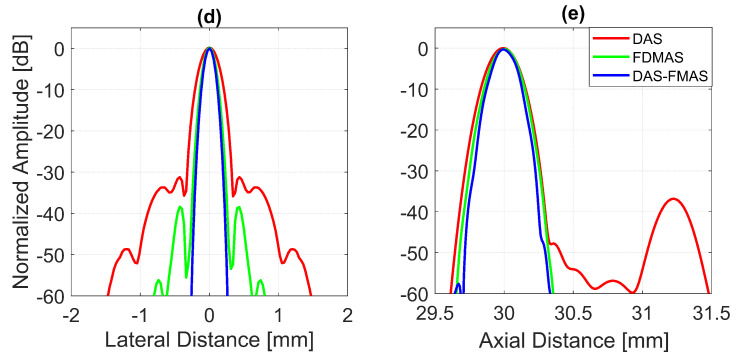
Single-point target compound PWI B-mode images beamformed with (**a**) DAS, (**b**) FDMAS and (**c**) DAS-FMAS, N = 3 ($-12^{\circ }$, $0^{\circ }$, $+12^{\circ }$). The lateral beam profile at a depth of 30 mm and the axial beam profile at x=0 mm are shown in (**d**,**e**) for all three beamforming techniques (DAS, FDMAS and DAS-FMAS).

To analyse in detail the effect of the proposed technique on a point target, B-mode images and beam profiles along the axial and lateral directions were plotted for a point target at a depth of 30 mm, as shown in Figure 8. It can be seen in Figure 8c that the side lobes along the lateral direction were nearly fully suppressed for an imaging dynamic range of 50 dB. However, this was not the case for DAS. With three compounding angles, the noise cancellation did not take place effectively. The ALs were still visible at approximately 31 mm of depth with DAS, as shown in Figure 8a. Although FDMAS was able to tackle the noise problem along the axial and lateral directions, the PSL produced along the lateral direction was higher than that with DAS-FMAS, which can be seen in Figure 8d. The PSL along the lateral direction at a depth of 30 mm for DAS was −31.9 dB, while for FDMAS it was −38.7 dB. For DAS-FMAS, the PSL along the lateral direction was reduced to −68 dB. The proposed technique, DAS-FMAS, produced narrower main lobes along the axial direction compared to DAS and FDMAS. This can be seen in Figure 8e. Complete AR measurements on the wire target at the 30 mm depth for DAS, FDMAS and DAS-FMAS (N=1 to N=25) are presented in the 13th figure (a,b) in Section 3.2.

### 3.2. Experimental Results

**Figure 9:**
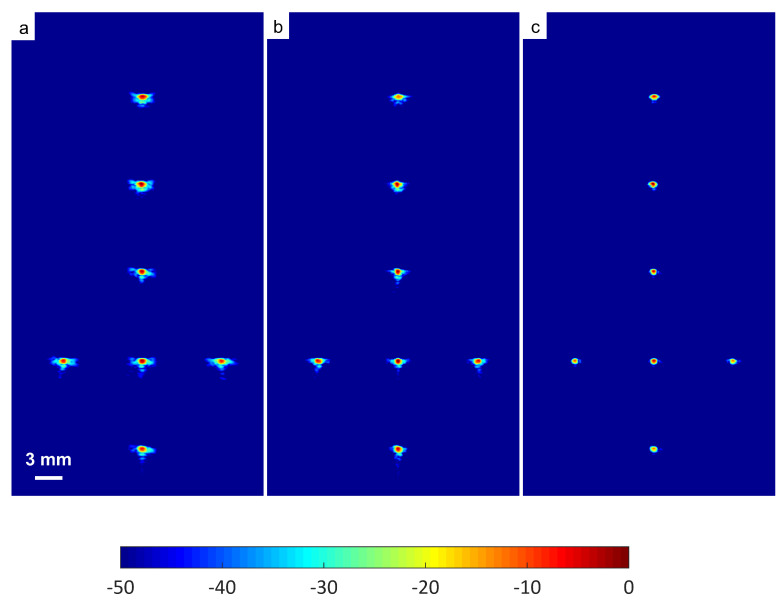

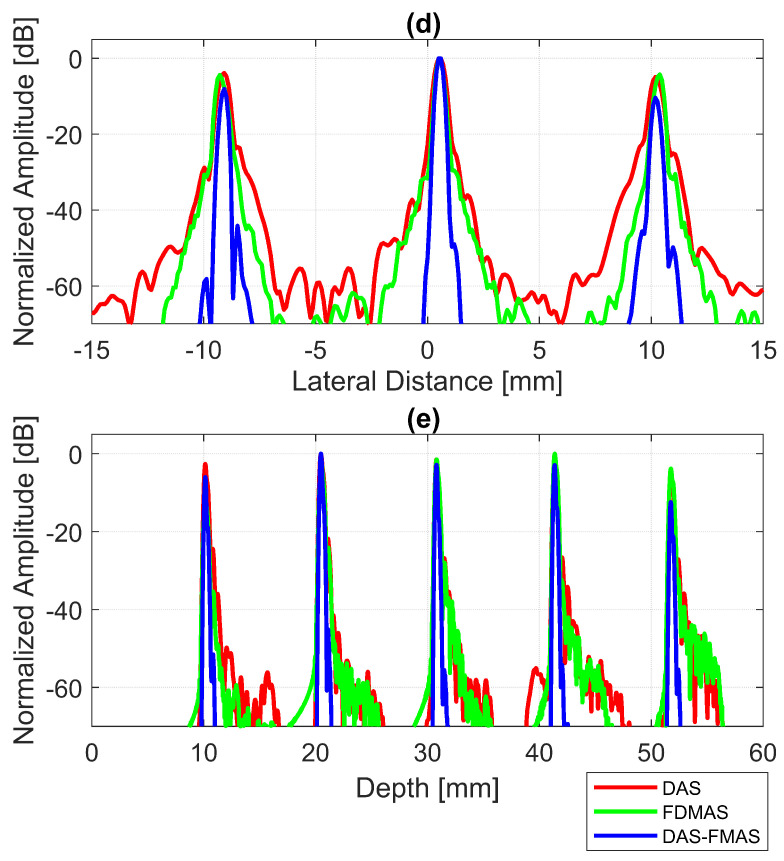
Wire phantom B-mode images were produced with N=13 for (**a**) DAS, (**b**) FDMAS and (**c**) DAS-FMAS. (**d**) Beam profiles along the lateral direction at 45 mm depth. (**e**) Axial beam profiles along x= 1.0 mm.

The experimental results on seven wire targets are presented in Figure 9. Thirteen steered plane waves, as given by Table 1, were used. A high number of compounding angles were able to eliminate the grating lobes in DAS. DAS-FMAS demonstrated a remarkable improvement since the side lobes along the axial and lateral axes were significantly reduced.

**Figure 10:**
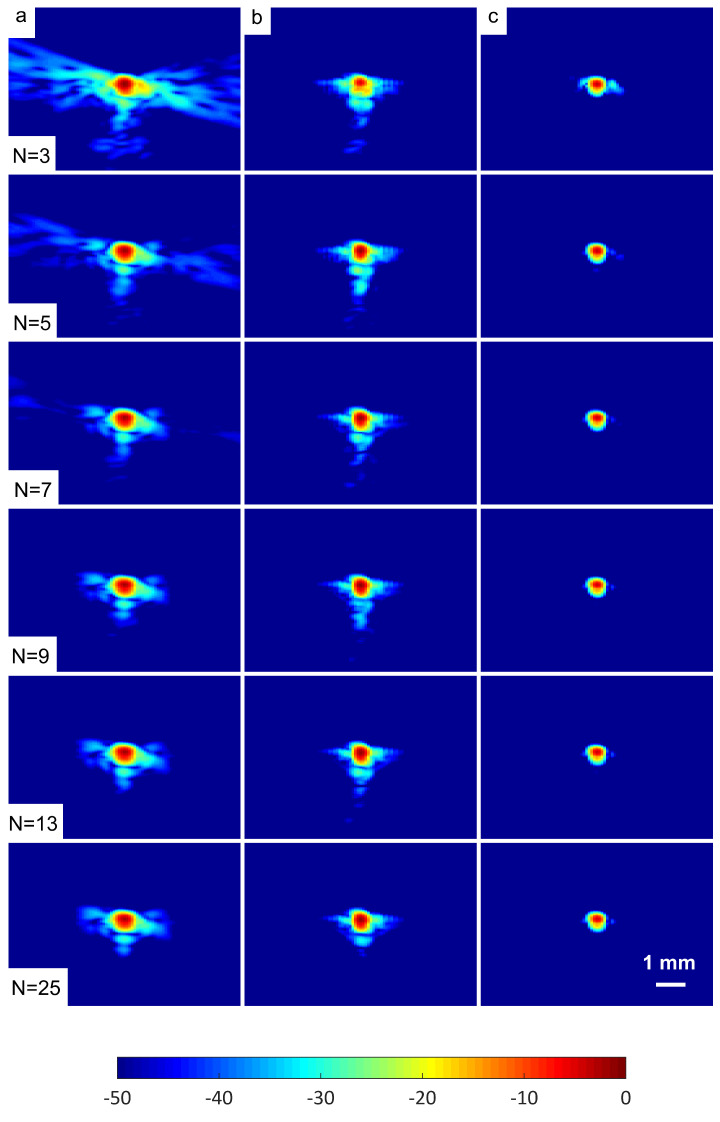
B-mode images with plane waves (N=3 to N=25) for the wire target at a depth of 30 mm beamformed with (**a**) DAS, (**b**) FDMAS and (**c**) DAS-FMAS. The side-lobe reduction in the lateral direction starts to improve with DAS-FMAS at the lowest number of compounding angles, N=3.

**Figure 11:**
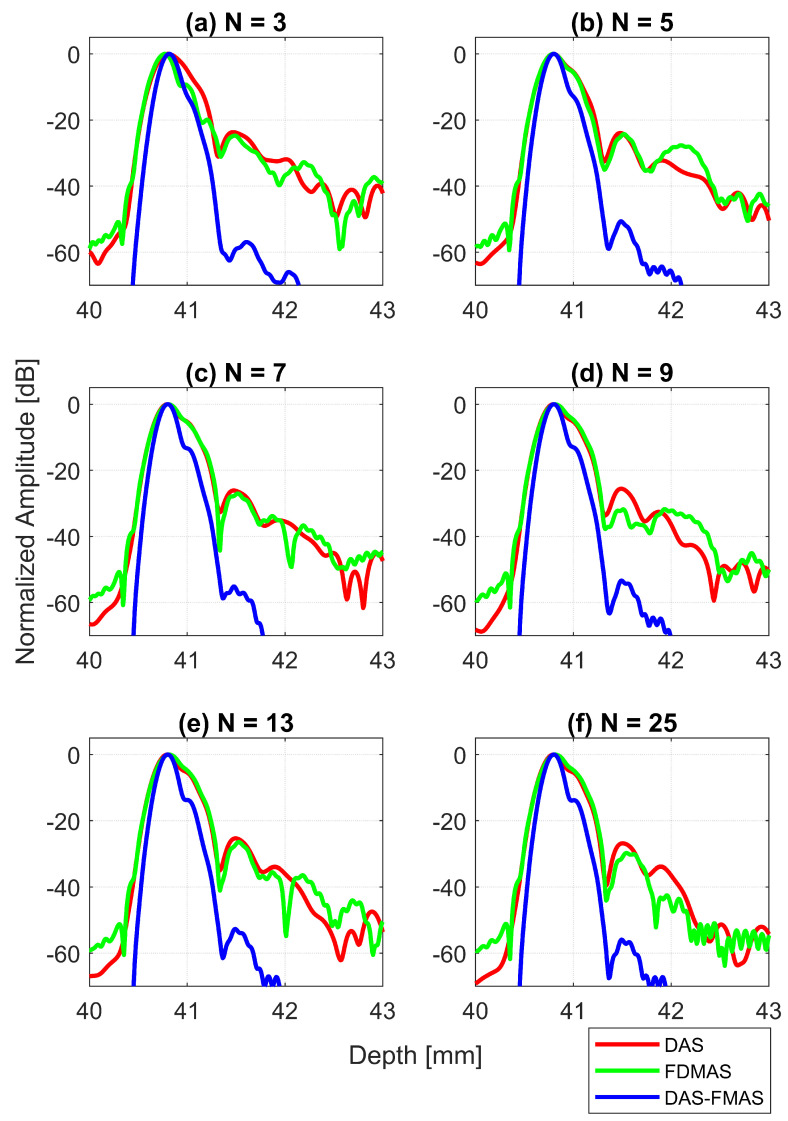
Axial beam profiles for the wire target at a 30 mm depth with DAS, FDMAS and DAS-FMAS using (**a**) N=3, (**b**) N=5, (**c**) N=7, (**d**) N=9, (**e**) N=13 and (**f**) N=25. There are no significant differences in axial lobes between DAS and FDMAS for all numbers of compounding angles, whereas for DAS-FMAS the main lobes are narrowed and the side lobes are attenuated by an average 25 dB.

**Figure 12:**
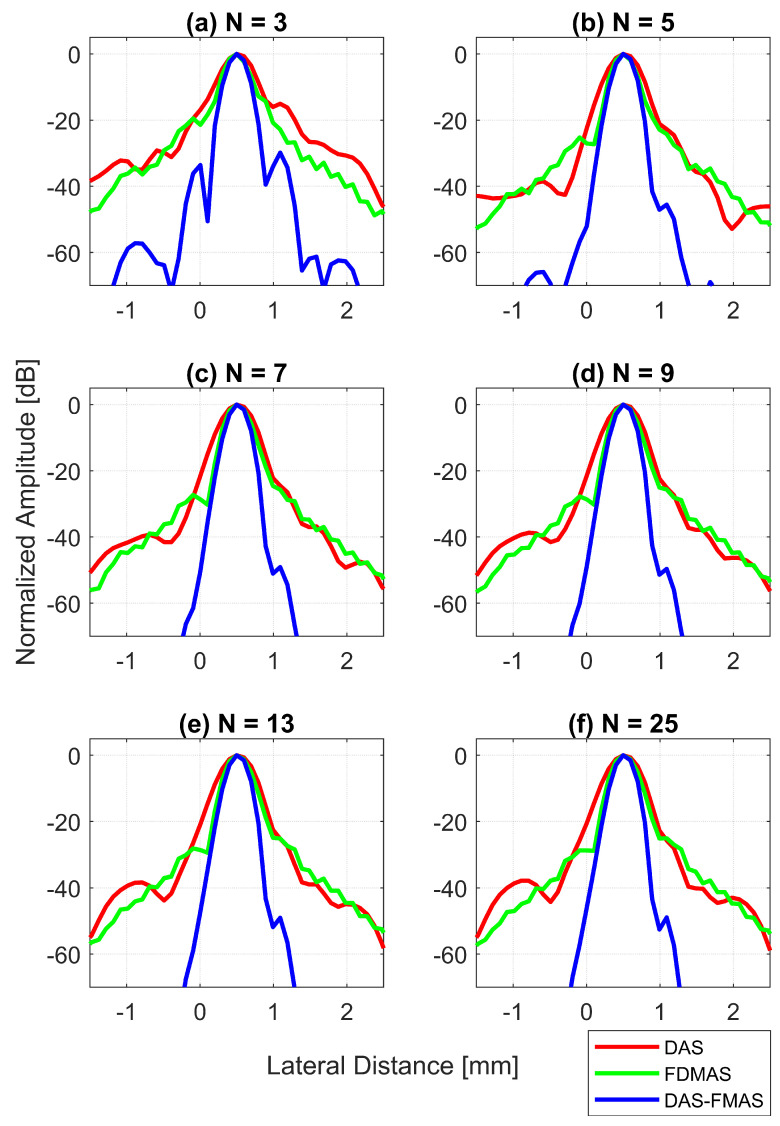
Lateral beam profiles for the wire target at a 30 mm depth with DAS, FDMAS and DAS-FMAS using (**a**) N=3, (**b**) N=5, (**c**) N=7, (**d**) N=9, (**e**) N=13 and (**f**) N=25. Comparing DAS-FMAS to DAS and FDMAS, the main lobe is narrower and the side lobes are attenuated more.

A wire target at a depth of 30 mm was selected in order to evaluate the spatial resolution of the DAS-FMAS method and compare it with conventional methods. Figure 10 shows B-mode images for N=3 to N=25 compounding angles for the wire target that were formed with DAS, FDMAS, and DAS-FMAS. The corresponding axial and lateral beam profiles for the wire target are given in Figure 11 and Figure 12, respectively.

**Figure 13:**
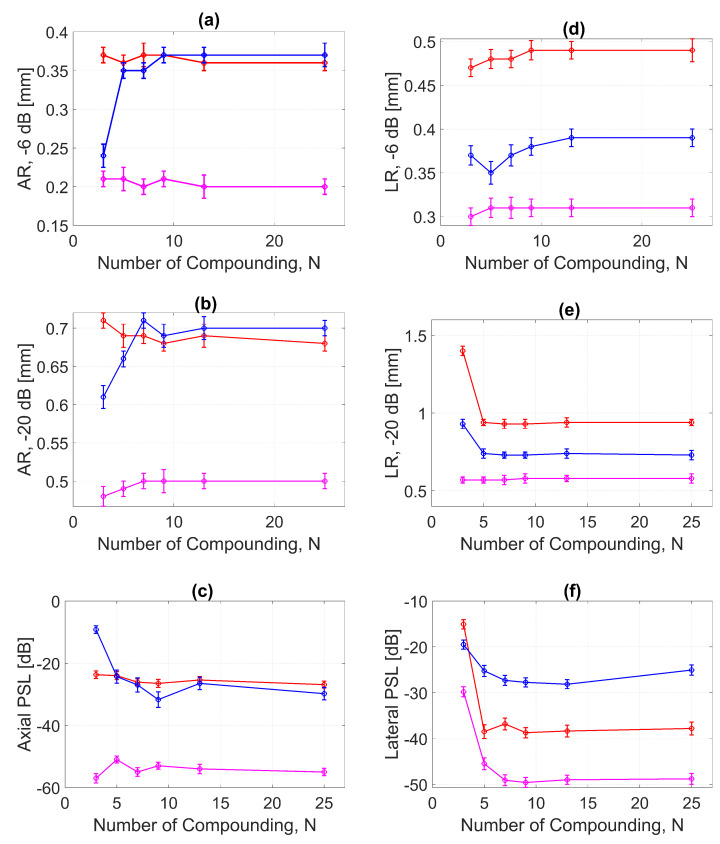
ARs for DAS, FDMAS and DAS-FMAS at the (**a**) −6 dB and (**b**) −20 dB levels were measured at a depth of 30 mm on the wire target. The PSL along the axial direction is presented in (**c**). The LR for DAS, FDMAS and DAS-FMAS at (**d**) −6 dB and (**e**) −20 dB measured at a 30 mm depth. The PSL along the lateral direction is presented in (**f**). Red, blue, and magenta color lines represent DAS, FDMAS, and DAS-FMAS, respectively.

The AR results for DAS, FDMAS and DAS-FMAS at the FWHM, −6 dB level were progressively more consistent with a compounding angle increase from N=3 to N=25. The AR improved significantly with DAS-FMAS compared to DAS and FDMAS. When N=3, the AR with DAS-FMAS was 43% and 12.5% better than that with DAS and FDMAS, respectively. When using 25 compounding angles, the AR improved by 44% and 47%, respectively, when compared to DAS and FDMAS. The ARs for DAS and FDMAS did not show any significant differences from N=5 to N=25 except for N=3. All results for the AR at −6 dB for different numbers of compounding angles are shown in Figure 13a. It was unexpected to see any improvement in AR for DAS and FDMAS through spatial compounding since its effect was in the lateral direction. This can be seen from the beam profiles along the axial direction, as shown in Figure 11 for the wire target at the 30 mm depth for all investigated techniques.

AR results at the −20 dB level for DAS, FDMAS and DAS-FMAS showed almost the same pattern as those at −6 dB. At N=3, the AR with DAS-FMAS was improved by 32% from DAS and 31% from FDMAS. While with 25 compounding angles, the AR was improved by 26% from DAS and 28.5% from FDMAS. The complete results for AR at −20 dB with all compounding angles are shown in Figure 13b.

With DAS-FMAS, the PSL in the axial direction was attenuated by 33 dB and 48 dB more than with DAS and FDMAS, respectively, for N=3. With 25 compounding angles, DAS-FMAS was able to reduce the PSL by 28 dB and 25 dB, respectively, more than DAS and FDMAS. All results for PSLs in the axial direction for different numbers of angles are shown in Figure 13c.

Figure 13d shows the LR results for DAS, FDMAS and DAS-FMAS at −6 dB. For all investigated techniques, a high LR was attained with fewer compounding angles, and DAS-FMAS yielded the best outcomes. In comparison to DAS and FDMAS, the LR for DAS-FMAS was enhanced by 36% and 19% for N=3. DAS-FMAS outperformed DAS and FDMAS by 37% and 20% when the number of compounding angles reached N=25. The LR for all approaches remained constant after N=13.

Figure 13e shows the LR results for DAS, FDMAS and DAS-FMAS at −20 dB. The LRs at −20 dB with N=3 for DAS, FDMAS and DAS-FMAS were 1.4 mm, 0.93 mm and 0.57 mm, respectively. DAS-FMAS outperformed DAS and FDMAS by 59% and 38%, respectively. With N=25, the LR with DAS-FMAS showed improvements of 38% and 20% compared to DAS and FDMAS, respectively. Beyond N=5, there were no changes in the LR at −20 dB for all techniques investigated.

The lateral PSL results for DAS, FDMAS and DAS-FMAS are presented in Figure 13f. As the steered plane waves increased from N=3 to N=25, the PSL results showed an improvement for all techniques investigated. DAS-FMAS produced the best outcomes in comparison to DAS and FMAS. At N=3, DAS-FMAS reduced the PSL by 14.7 dB and 10.3 dB more than DAS and FDMAS, respectively. When compared to DAS and FDMAS, the PSL with DAS-FMAS was 11.1 dB and 23 dB lower for N=25.

**Figure 14:**
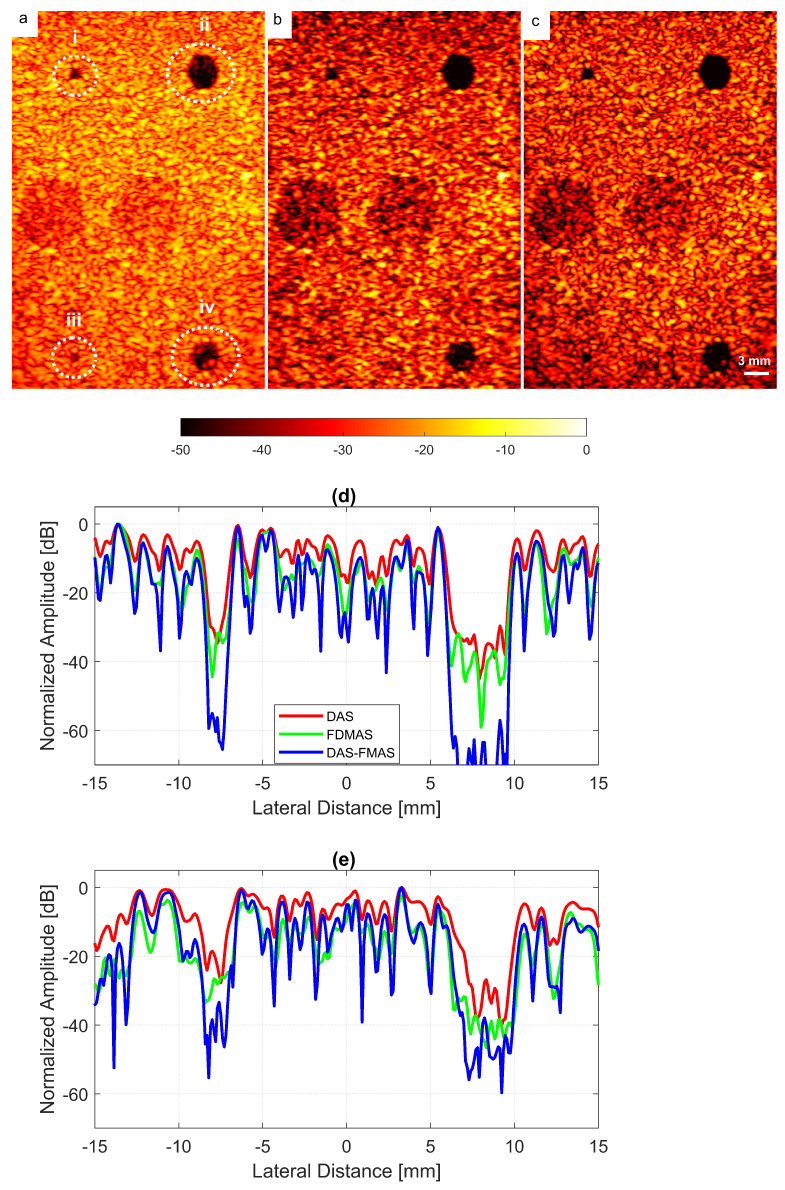
Cyst phantom B-mode images for (**a**) DAS, (**b**) FDMAS and (**c**) DAS-FMAS were produced with N=13. Beam profiles along the lateral direction at (**d**) 15 mm and (**e**) 45 mm depth. The two smallest circles (ii and iii) and the two largest circles (ii and iv) at 15 mm and 45 mm depth represent 1.3 mm and 3.0 mm diameter cysts, respectively.

The experimental results on cysts with diameters of 1.3 and 3.0 mm at depths of 15 and 45 mm with 13 compounding angles as given in Table 2 are shown in Figure 14a–c. FDMAS and DAS-FMAS improved the CRs for all cysts in circles i, ii, iii and iv as compared to DAS. When compared to DAS and FDMAS, DAS-FMAS reduced more clutter noise. This can be seen on the B-mode image of the 1.3 mm diameter cyst (marked as circle iii) which was barely visible with DAS and FDMAS but the contrast was improved with DAS-FMAS. The lateral beam profiles at 15 mm and 45 mm are shown in Figure 14d and Figure 14e, respectively.

**Figure 15:**
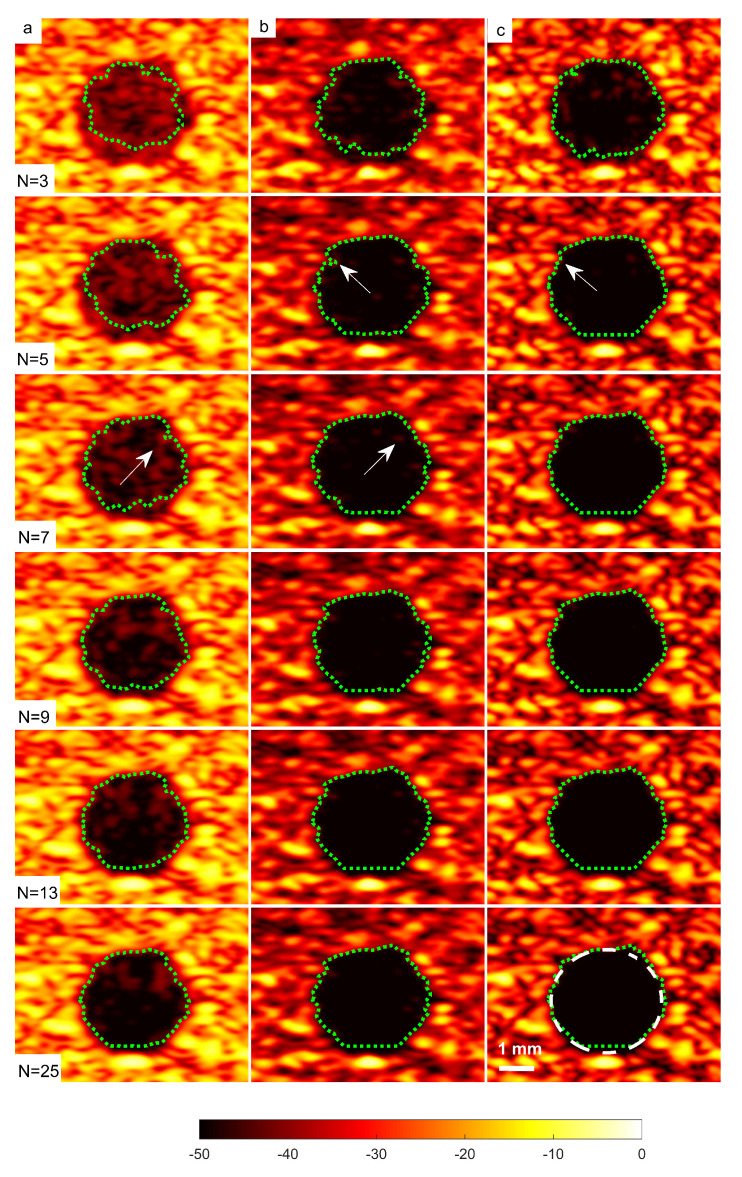
A set of B-mode images for a cyst with a diameter of 3.0 mm located at a depth of 15 mm using (**a**) DAS, (**b**) FDMAS and (**c**) DAS-FMAS with N=3 to N=25 steered plane waves. The green dashed line shows the BSAC implementation. The arrows on N=5 images with FDMAS and DAS-FMAS highlight the side-lobe reduction, and in N=7 images highlight the clutter noise with DAS and FDMAS that is no longer present inside the cyst with DAS-FMAS. The white dashed circle on (**c**), N=25, shows the segmentation ground truth for the 3.0 mm diameter cyst to calculate the Dice coefficient and MIoU.

**Figure 16:**
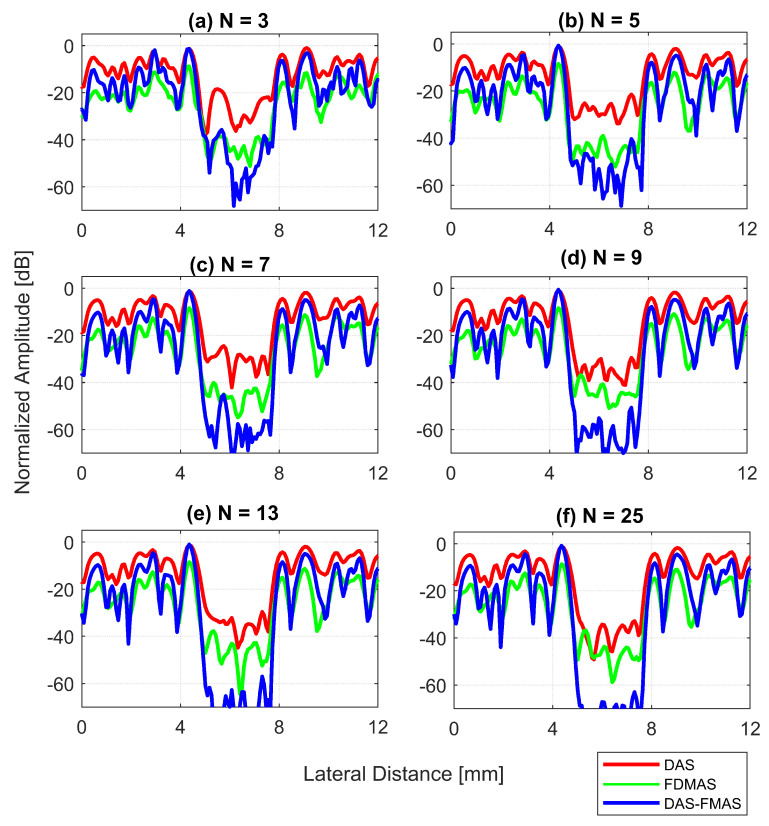
Lateral beam profiles for cysts with a diameter of 3.0 mm at a depth of 15 mm for (**a**) N=3, (**b**) N=5, (**c**) N=7, (**d**) N=9, (**e**) N=13 and (**f**) N=25.

A cyst with a 3 mm diameter located at a 15 mm depth as marked by circle ii in Figure 14 was chosen to measure the images’ CR and CNR. The B-mode images for the cysts with DAS, FDMAS and DAS-FMAS are shown in Figure 15a, Figure 15b and Figure 15c, respectively. All images are displayed with a 50 dB dynamic range. In general, FDMAS and DAS-FMAS performed better than DAS, where more clutter noise was reduced inside the anechoic region. The beam profile along the lateral direction at the 15 mm depth is given in Figure 16. When the number of steered plane waves increased from N=3 to N=25, the clutter noise level of 3.0 mm diameter cysts kept decreasing.

Figure a shows the CR results for the 3.0 mm diameter cyst at the 15 mm depth. As the number of compounding angles increased, the CRs for all techniques continued to increase. With N=3, DAS-FMAS outperformed DAS and FDMAS in CR by 14.1 dB and 7.29 dB, respectively. The CR for DAS-FMAS was −49.8 dB with N=25, which was higher than that for DAS (−26.1 dB) and FDMAS (−27.9 dB). Figure 16 illustrates that DAS-FMAS could eliminate clutter noise within anechoic regions by attenuating it to a level lower than −60 dB.

**Figure 17:**
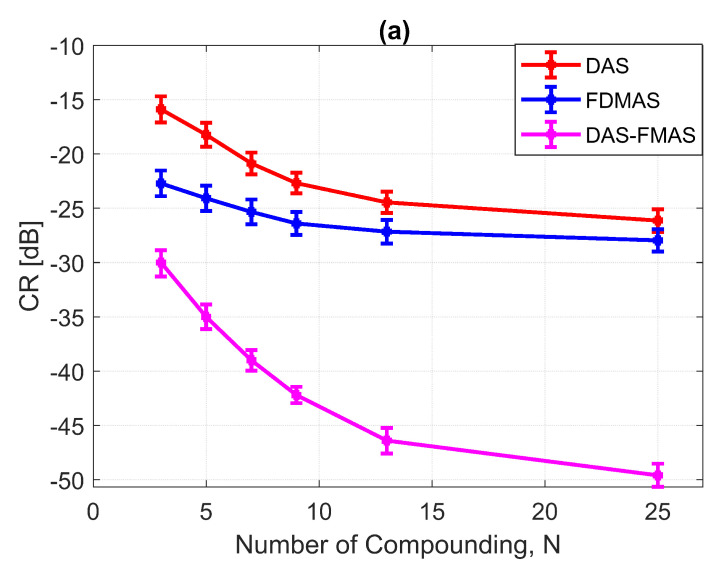

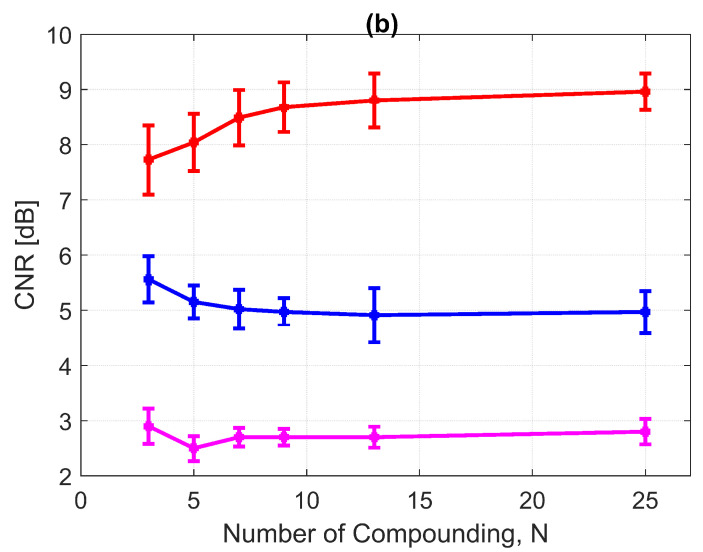
(**a**) CRs and (**b**) CNRs for a 3.0 mm diameter cyst at 15 mm depth employing DAS, FDMAS and DAS-FMAS.

The CNRs for the 3.0 mm diameter cyst at a 15 mm depth are given in Figure 17b. As opposed to all other performance indices, the CNR for DAS-FMAS was the lowest compared to those using DAS and FDMAS. The CNR did not show significant variations for DAS-FMAS from N=3, 2.9 dB to N=25, 2.8 dB. The CNR for FDMAS kept decreasing for the same compounding range of N=3 to N=25, while for DAS the CNR kept increasing. The reduction of clutter noise outside the cyst reduced the CNR for DAS-FMAS. The destructive speckle regions in DAS were filled with clutter noise. Once the clutter noise was reduced, the destructive region became more visible as the dark spot. This can be seen from the beam profile shown in Figure 16. Outside the cyst regions, the speckle variation was higher with FDMAS and DAS-FMAS. One of the ways to solve the low CNR problem was by using despeckling, which reduced the speckle fluctuation.

The clutter noise reduction at a 45 mm depth for all techniques was less than that at a 15 mm depth. This was mainly due to the low SNR at deeper locations. DAS-FMAS still performed better than the other two techniques, even at the deeper location. The level of clutter noise inside the 3.0 mm diameter cyst continued to decrease as the number of steered plane waves increased from N=3 to N=25.

### 3.3. Effects of Different Beamforming Techniques on Segmentation

The sizes of the cyst areas of the CIRS phantom, as highlighted by green dashed lines in Figure 15, were measured and compared to their nominal values. The region size was calculated by counting the pixels within the cyst and multiplying them by their axial and lateral pixel sizes. The B-mode image pixel size along the axial direction was calculated to be 9.625 μm. With a diameter of 3.0 mm, the nominal cyst area was 7.07 mm${}^{2}$. The measured cyst sizes approximated their nominal values when the number of steered plane waves increased from N=3 to N=25 for all techniques investigated, as shown in Table 3. However, DAS-FDMAS was able to achieve almost the exact nominal value with a low number of compounding angles, N=5, compared to the other two techniques. This showed the new compounding technique improved the BSAC-based segmentation process. However, the cyst size measured with DAS and N=25 still differed from the nominal value by 0.22 mm${}^{2}$. For cysts with a diameter of 3.0 mm, the time for the snake to reach the segmented cyst border, represented by the contour shown in green in Figure 15, was computed by setting the number of iterations to 100. Depending on how noisy the object is, a snake’s total convergence time from the object’s centre to the intended border will vary. If the snake is unable to reach its minimal energy due to high clutter noise, it may take longer. Table 3 shows the time it took for the snake to converge to the 3.0 mm cyst boundaries with DAS, FDMAS and DAS-FMAS.

In comparison to FDMAS and DAS-FMAS, the snake convergence time with DAS was much longer for all numbers of compounding angles. All of the investigated methods demonstrated that the time it took for the snake to converge decreased as the number of steered plane waves increased. It also demonstrated that reducing clutter noise within the cyst region allowed the snake to converge on the cyst boundary faster, and DAS-FMAS helped provide a more accurate segmentation and estimation of the cyst size when compared with FDMAS.

Table 4 shows the results for the Dice coefficient and MIoU with DAS, FDMAS and DAS-FMAS. In general, the results of all investigated methods for Dice and MIoU indices improved as the number of compounds increased from N=3 to N=25. Both FDMAS and DAS-FMAS outperformed DAS. Starting from N=5, the high Dice coefficient and MIoU values obtained with the DAS-FMAS compounding technique indicated that the predicted segmentation area was approaching the ground truth. The main reason for this achievement was the reduction of clutter noise, which allowed the contour to evolve to actual cyst borders.

### 3.4. In Vivo Images

**Figure 18:**
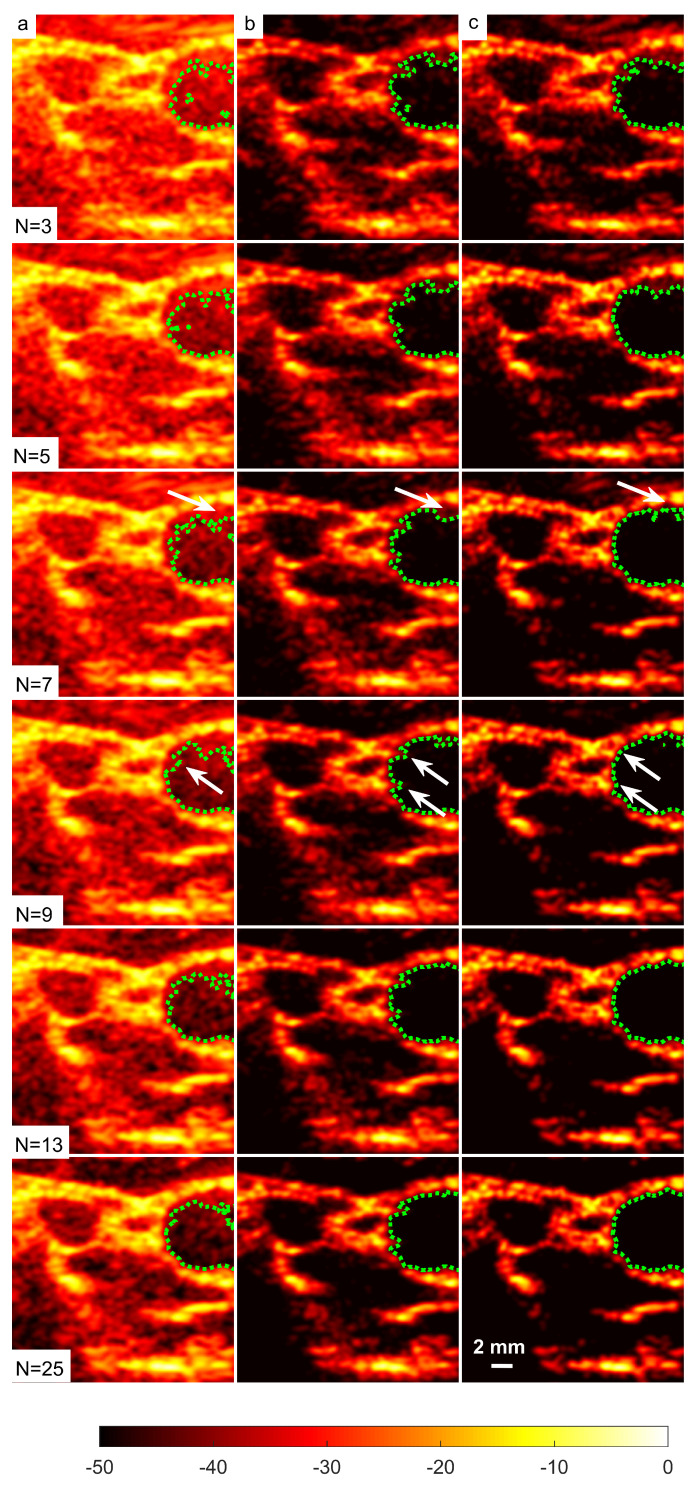
Right-side CCA B-mode images produced with (**a**) DAS, (**b**) FDMAS and (**c**) DAS-FMAS from N=3 to N=25, and their corresponding segmentation results.

The in vivo B-mode images obtained from DAS, FDMAS and DAS-FMAS are presented in Figure 18. All the images are shown with a 50 dB dynamic range. The suppression of clutter and noise when using FDMAS and DAS-FMAS was noticeable on the B-mode images starting with N=3. When the number of steered plane waves increased to N=25, DAS-FMAS suppressed noise and clutter in the common carotid artery (CCA) and the nearby anechoic region. The side lobes in the CCA were still visible with FDMAS. In contrast, the vast majority of the imaging region using DAS was still dominated by clutter noise. Clutter noise reduction and spatial and contrast resolution enhancement in the CCA region facilitated a better segmentation. The arrows in N=9 images with DAS and FDMAS indicate the side lobe leaking into the CCA anechoic regions. When the carotid border was being segmented, such leaks became an obstacle to the formation of contours. The segmentation procedure was complicated by the existence of clutter noise artefacts in the ultrasound B-mode image, which prevented BSAC from converging on the desired border. Thus, employing DAS-FMAS to reduce side lobes and clutter noise in the CCA anechoic region enhanced the segmentation process overall. Furthermore, the clutter noise region (highlighted by arrows on Figure 18) impairing the segmentation process with DAS and FDMAS could be falsely identified as a plaque. The corresponding inaccurate diagnosis could lead to improper treatment. A good segmentation output is also necessary for the three-dimensional reconstruction of the CCA from two-dimensional transversal ultrasound B-mode imaging, and it is anticipated that the DAS-FMAS image-based segmentation could also benefit this application.

## 4. Conclusions

In this paper, we addressed in depth a new compounding technique that we recently developed, which was based on the autocorrelation technique. Recently, many researchers have adopted our method for their research, but none of them have addressed the theory underlying the enhancement of the ultrasound image’s AR, which was the primary contribution of this technique. The autocorrelation process and the phase misalignment produced by angular steered plane waves were the two key factors contributing to the capability of the proposed method to increase the quality of the B-mode image. Although the proposed compounding technique can also produce a better LR and CR, in this work, the enhancement that took place on the AR was discussed in detail. This was to emphasize the uniqueness of the proposed method to improve the AR without employing higher-bandwidth excitation signals such as a chirp or a short square pulse that are bound by many restrictions. Additionally, we discussed a biomedical application of the proposed method in this study, showing how the BSAC technique performed better when segmenting the common carotid artery on the B-mode image produced by the proposed technique. Our future research will make use of this method to measure intima-media thickness, one of the most important markers for detecting cardiovascular disease.

## Figures and Tables

**Table 1:** Compounding parameters.

Properties	Values					
No. of compounding, *N*	3	5	7	9	13	25
Angle increment, Δθ	12	6	4	3	2	1

**Table 2:** Simulation and experimental parameters.

Properties	Values
Speed of sound in water/CIRS phantom, m/s	1482/1540
Attenuation in water/CIRS, dB/MHz/cm	0.002/0.5
Number of elements	128
Transducer centre frequency, MHz	4.79
Transducer bandwidth (−6 dB), %	57
Transducer element pitch, mm	0.3048
Sampling frequency, Tx/Rx, MHz	160/80
Excitation	2-cycle sinusoid

**Table 3:** Segmented region size and snake convergence time for 100 iterations.

N	DAS		FDMAS		DAS-FMAS	
	**Area, mm** ${}^{2}$	**Time, s**	**Area, mm** ${}^{2}$	**Time, s**	**Area, mm** ${}^{2}$	**Time, s**
3	5.95 ± 0.04	1.71 ± 0.01	6.73 ± 0.05	0.06 ± 0.003	6.92 ± 0.04	0.06 ± 0.002
5	6.18 ± 0.02	0.55 ± 0.02	6.92 ± 0.03	0.03 ± 0.002	7.03 ± 0.03	0.02 ± 0.002
7	6.29 ± 0.03	0.55 ± 0.02	6.93 ± 0.02	0.03 ± 0.001	7.04 ± 0.01	0.02 ± 0.001
9	6.45 ± 0.04	0.54 ± 0.01	7.01 ± 0.02	0.03 ± 0.002	7.04 ± 0.02	0.02 ± 0.002
13	6.83 ± 0.03	0.54 ± 0.02	7.01 ± 0.01	0.02 ± 0.001	7.04 ± 0.02	0.02 ± 0.003
25	6.85 ± 0.02	0.52 ± 0.02	7.02 ± 0.02	0.02 ± 0.001	7.06 ± 0.02	0.02 ± 0.002

**Table 4:** BSAC segmentation performance.

N	DAS		FDMAS		DAS-FMAS	
	**MIoU**	**Dice**	**MIoU**	**Dice**	**MIoU**	**Dice**
3	0.841	0.913	0.951	0.974	0.979	0.988
5	0.872	0.932	0.979	0.988	0.993	0.996
7	0.890	0.941	0.981	0.990	0.995	0.998
9	0.911	0.954	0.990	0.995	0.995	0.998
13	0.963	0.981	0.991	0.995	0.995	0.998
25	0.964	0.984	0.992	0.995	0.999	0.999

## Data Availability

Not applicable.
